# Vibration-Based Loosening Detection of a Multi-Bolt Structure Using Machine Learning Algorithms

**DOI:** 10.3390/s22031210

**Published:** 2022-02-05

**Authors:** Oybek Eraliev, Kwang-Hee Lee, Chul-Hee Lee

**Affiliations:** 1 Future Vehicle Engineering Department, Inha University, 100 Inharo, Mitchuholgu, Incheon 22212, Korea; oybekeraliev7@gmail.com; 2 Mechanical Engineering Department, Inha University, 100 Inharo, Mitchuholgu, Incheon 22212, Korea; gwanghee.yee@gmail.com

**Keywords:** bolt loosening, loosening detection, machine learning, bolt-loosening identification, vibration, signal processing

## Abstract

Since artificial intelligence (AI) was introduced into engineering fields, it has made many breakthroughs. Machine learning (ML) algorithms have been very commonly used in structural health monitoring (SHM) systems in the last decade. In this study, a vibration-based early stage of bolt loosening detection and identification technique is proposed using ML algorithms, for a motor fastened with four bolts (M8 × 1.5) to a stationary support. First, several cases with fastened and loosened bolts were established, and the motor was operated in three different types of working condition (800 rpm, 1000 rpm, and 1200 rpm), in order to obtain enough vibration data. Second, for feature extraction of the dataset, the short-time Fourier transform (STFT) method was performed. Third, different types of classifier of ML were trained, and a new test dataset was applied to evaluate the performance of the classifiers. Finally, the classifier with the greatest accuracy was identified. The test results showed that the capability of the classifier was satisfactory for detecting bolt loosening and identifying which bolt or bolts started to lose their preload in each working condition. The identified classifier will be implemented for online monitoring of the early stage of bolt loosening of a multi-bolt structure in future works.

## 1. Introduction

A bolt joint is one of the most important methods for connecting structural components in engineering fields and can be assembled and reused. However, one of the main drawbacks of threaded fasteners is the loosening that occurs under shock or vibration conditions [1]. The loosening can cause a structure to become damaged seriously, so bolt-loosening detection and identification before failure of the structure are some of the most important issues in engineering. Detection and identification of bolt loosening can, not only keep a structure from experiencing accidents or failure, but also reduce maintenance costs. Therefore, the detection of bolt loosening in many fields of mechanical, aerospace, and civil engineering has been an important topic among many researchers in the last decade.

Generally, the detection techniques for bolt loosening can be divided into three groups. The first is the group of in situ inspection techniques, which include visual inspection by an experienced inspector or use of mechanical devices such as a torque wrench and hammer [2]. Despite the advantages of visual inspection by an expert, which is the simplest and lowest-cost method, it is complicated to detect the early-stages of bolt loosening. In other words, the visual inspection method is better as a way to detect bolts which are totally loosened. However, the hammer impact method is more effective to detect early-stage of bolt loosening than visual inspection, and bolt loosening can be detected easily via the sound that is made when hitting something with a hammer by an experienced inspector or using ML algorithms [3]. Although the group of in situ inspection techniques are quite simple and low-cost monitoring techniques, they still have challenges [4]. For instance, one of the main drawbacks of the hummer impact technique is that environmental noise can reduce the accuracy, and the technique is time consuming in some applications that include many bolts, such as bridges [2].

The second group includes computer vision-based techniques that detect bolt-loosening by use of cameras or digital images [5,6,7,8]. This group of techniques is unique, with advantages that can overcome the drawbacks of the in situ inspection techniques such as environmental noise and being time consuming. Many researchers have contributed to the development of computer vision-based techniques to detect bolt loosening. For example, Zang et al. [9] carried out a study on bolt-loosening detection using deep learning for a multi-bolt connection. Their model solves two problems: (1) a bolt detection with a convolutional neural network (CNN), and (2) a regression problem to predict the amount loosening of the bolt with a faster region-based convolutional neural network (Faster R-CNN).

Bolt loosening was detected by measuring the rotation angle of a bolt or nut via deep learning in a study by Zhao et al. [10]. They used a set of images taken with a smartphone as a dataset for detection of a bolt head and a number that was written on top of the bolt. The coordinates of the detection boxes of the bolt head and number were used to find the center points of the boxes, and the rotation angle of the bolt was computed using the center points. Valuable investigations have been performed by researchers with this technique. However, there are still some challenging issues. For example, detecting the early stages of the loosening of a bolted joint is very difficult, and in some real conditions, such as those in vehicle engines and turbines, fixing a camera to an appropriate place to detect loosening is complicated. A CNN is also often used as a tool in this technique and has a high computational cost.

The third group includes sensor-based techniques, which include the vibration-based method [11,12], acoustoelastic effect-based method [13,14], piezoelectric sensor-based methods [15,16], and impedance-based method [17,18]. Sensor-based techniques have some unique advantages that can overcome the drawbacks of in situ inspection and computer vision-based techniques such as environmental noise impact, being time consuming, as well as the detection of early-stage bolt loosening. However, sensor-based techniques require fixed sensors and a high-cost system, while the accuracy of the techniques is significantly greater than that of the other techniques that have been described above, and they are reliable [2]. Computer vision-based techniques for bolt loosening detection of multi-bolt connections have been proposed by several researchers, while a few studies based on vibration-based methods, with use of ML algorithms, for early stage bolt loosening detection and identification have been carried out [19].

Therefore, one of the most popular sensor-based techniques, a vibration-based method, was chosen in this study. This paper proposes the classification and identification of early stages of bolt loosening in a multi-bolt structure using ML classifiers. The application is an AC Motor. For this, 16 cases (healthy and unhealthy conditions) were established, and vibration data were gathered in three operating conditions of the motor (800 rpm, 1000 rpm, and 1200 rpm). A short-time Fourier transformation was conducted to obtain features of the row vibration data. Finally, ML classifiers were evaluated to determine the best classifier, and the classifier that had the greatest accuracy was identified.

The results prove that the determined classifier can detect, not only the early-stages of loosening, but can also identify which bolt or bolts have started to loosen. The theoretical background of signal processing, feature extraction of vibration data for ML classifiers, and ML algorithms are described in Section 2. Section 3 explains the experimental setup in detail. The results and contributions of the study are discussed in Section 4. The conclusions are presented in Section 5.

## 2. Theoretical Background and Signal Processing

**Figure 1:**
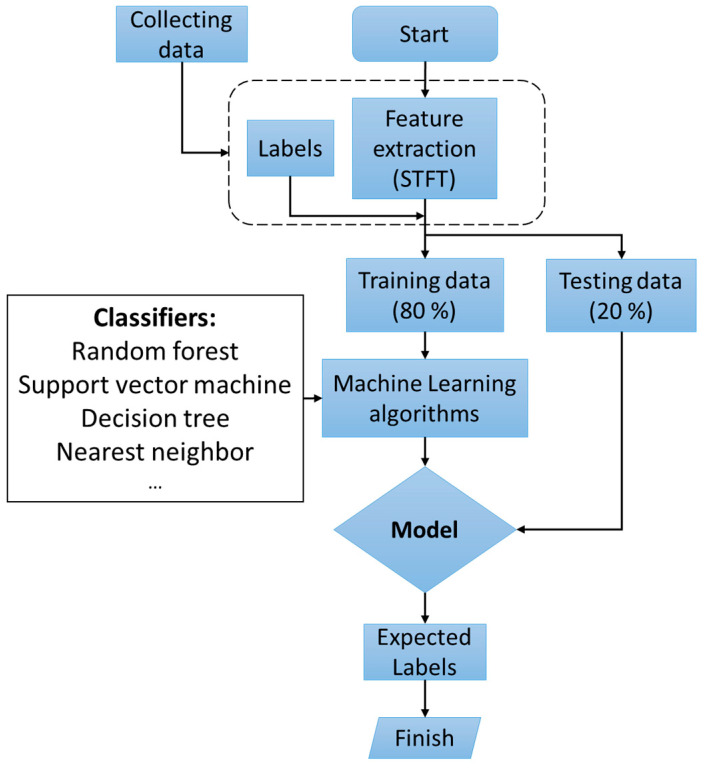
The flowchart of the proposed method for bolt-loosening detection.

A flowchart of the method proposed in this study is shown in Figure 1. Initially, data acquisition is performed on a motor that has four M8 bolts. Then, short-time Fourier transform (STFT) is applied to extract features of the acquired vibration data. Following this, the extracted dataset is divided into a training dataset for training a model of classifiers and a test dataset for evaluating the accuracy of the models. Finally, ML classifiers are evaluated to determine the best classifier, and the classifier that has the best performance is identified.

### 2.1. Feature Extraction (STFT)

Feature extraction can play a key role in obtaining a reliable result in the ML field. Feature extraction of a row vibration signal can be done in several ways. For example, the fast Fourier transform (FFT) method computes the discrete Fourier transform (DFT) of a data sequence. Fourier analysis transforms a signal from its original domain (typically time or space) to a frequency domain representation, or conversely [20]. Chen et al. [21] used a FFT method in their study for identification and classification of a gearbox fault. S. Ma et al. [22] also used FFT in their study for fault diagnosis of a rotor and bearing. However, performing the FFT on a time-domain signal can give the overall frequency components for the entire time-domain signal. Therefore, it cannot provide information about how the frequency is changing over time. To understand, for example, where the high and low pitches are in a vibration signal, the STFT can be applied. It can give a FFT that changes with time. This is not readily apparent from only applying the FFT to the entire time-domain signal, as this gives one set of components that are not time dependent [23,24]. Hence, the STFT method is commonly used in feature extraction of a vibration signal [25,26,27]. A time-domain signal is converted into a time-frequency-domain signal in the STFT method. It splits up the long time-domain signal into several segments by use of the same size of window function, and FFT coefficients are calculated for the segments. The calculated values, corresponding to the segments, are stored as a matrix. One of the advantages of STFT is that the output of the STFT can be directly used for the training of classifiers. The number of windows are used as training examples, and FFT coefficients for a window are used as features. Therefore, STFT is used for feature extraction of vibration data in this study.

The mathematical function of STFT can be expressed as:

(1) $$STFT\left(\tau ,w\right)=\overset{\infty }{\underset{-\infty }{\int }}s\left(t\right)w\left(t-\tau \right)e^{-j\omega t}dt$$

where s(t) is the original vibration signal, w(t) is a windowing function, t is time, and τ is time index. There are several windowing functions for STFT, such as rectangular, triangular, Hamm, Kaiser, Blackman, Gaussian, and Hann functions. The Hann windowing function is usually a good choice and is frequently employed with random data because, when compared to the impacts of other windows, it has a moderate impact on the frequency resolution and amplitude accuracy of the resulting frequency spectrum [28]. Therefore, the Hann windowing function was used in this study and can be expressed as follows:

(2) $$h\left(t\right)=\{\begin{array}{c} 0.5\left[1-\cos\left(\frac{2\pi n}{M-1}\right)\right],\;0\leq n\leq M-1\\ 0,\;otherwise \end{array}$$

where n is time index and M is the number of samples.

STFT is performed for the vibration signal using the librosa open-source library in Python. The output is an $n_{n\_fft}\times m_{s}$ matrix, which contains complex numbers, and absolute values of the output are used. Here, n_fft is the number of FFT coefficients for each window (n_fft = 1024), and $m_{s}$ is the number of windows. $n_{n\_fft}$ is calculated as follows:

(3) $$n_{n\_fft}=\frac{n\_fft}{2}+1$$

The hop size is 512. After feature extraction, the data were divided into 80% training data for training the model of classifiers and 20% test data for evaluating the models. Each experiment was carried out 20 times, and average values were used for the analysis.

### 2.2. Machine Learning

Since humans evolved, numerous types of tools have been used to fulfill different kinds of task in the easiest way. Different machines have been invented by humans for various tasks in human life, such as industry, computing, and so on. ML is one of them.

ML algorithms are implemented to teach machines how to handle data more fruitfully. Sometimes, by looking at the data, it is difficult to interpret the information. ML can help in this case. ML algorithms learn key features and the pattern of data, and based on this, they predict a new value. ML can be divided into several groups, such as supervised learning, unsupervised learning, semi-supervised learning, reinforcement learning, multi-task learning, ensemble learning, neural networks, and instance-based learning [29]. This study belongs to supervised learning. Some popular ML classifiers used in this study are briefly described below.

The Decision Tree Algorithm is part of the supervised machine learning family of algorithms. It is applicable to both classification and regression problems. The purpose of this algorithm is to develop a model that predicts the value of a target variable, and the decision tree solves the problem by using the tree representation, where the leaf node corresponds to a class label and characteristics are represented on the internal node of the tree [29]. The Support Vector Machine (SVM) is a part of the supervised machine learning models with accompanying learning algorithms that examine the data used for classification and regression analysis. SVM can perform non-linear classification, as well as linear classification, by implicitly mapping inputs into high-dimensional feature spaces, which is known as the kernel trick. Basically, this is used to draw lines between classes. The margins are drawn so that the space between the margin and the classes is as small as possible, reducing the classification error [30]. The K-Nearest Neighbor (KNN) algorithm is a simple supervised machine learning technique that can be used to handle classification and regression problems. It is simple to set up and comprehend, but it has the problem of being noticeably slower, as the amount of data in use grows [30]. The Random Forest algorithm is an assembled approach that generates trees using a CART (classification and regression trees) methodology to a maximum size and without pruning [31]. Bagging or Bootstrap aggregating is used when the accuracy and stability of a machine learning algorithm need to be improved. It can be used for both classification and regression. Bagging also reduces variation and aids in the management of overfitting of a decision tree [30]. XGBoost is a scalable machine learning system for tree boosting that was proposed by Chen and Guestrin in 2016 [32]. XGBoost is a well-known machine learning method that consistently outperforms other machine learning algorithms. Indeed, it has evolved into the ‘state-of-the-art’ machine learning technique for dealing with structured data since its debut. [33]. The Linear Discriminant Analysis (LDA) algorithm is a frequently used classification technique. The LDA method works by calculating the variance values within and between classes [34].

## 3. Experimental Setup

**Figure 2:**
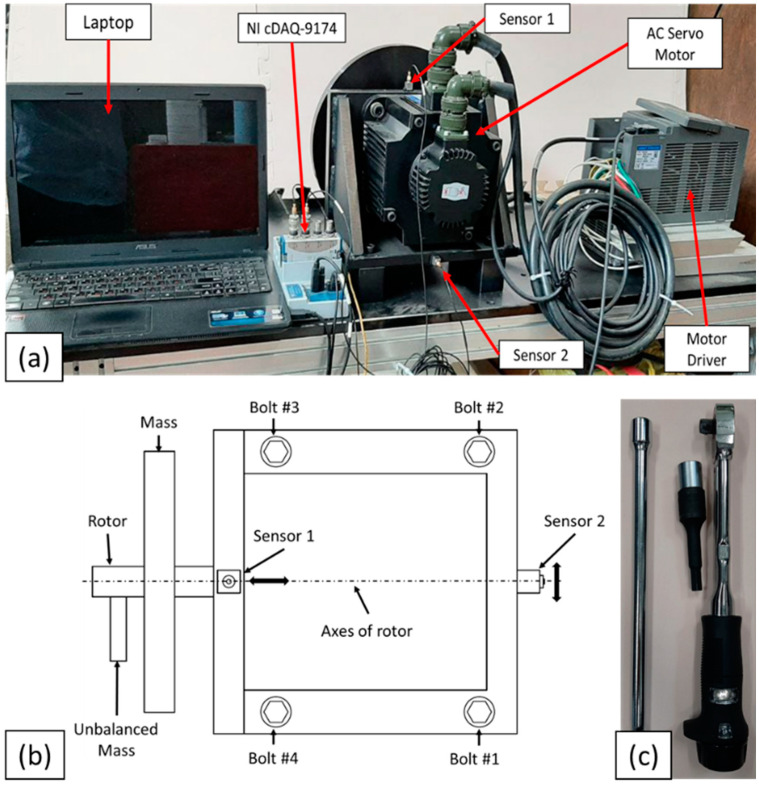
(**a**) Experimental setup; (**b**) scheme of top view of motor and position of bolt specimens and sensors; (**c**) torque wrench.

This study proposes a vibration-based method of early-stage bolt loosening detection and identification combined with an ML classifier for a multi-bolt structure. The experimental setup is shown in Figure 2a. The application in this study is an AC Motor, and the specification of the motor is highlighted in Table 1. The motor was fastened with four M8 × 1.5 steel bolts (property class 8.8). The position of the bolts is shown in Figure 2b. According to the property class of the bolt, the maximum torque load for the M8 × 1.5 bolt is 27.5 N·m. Therefore, a 25 N·m torque load was considered a tightened (healthy) condition, and the torque of 19 N·m was used as a loosened (unhealthy) condition. When the decrement of a torque load is less than 6 N·m (for example, 4 or 5 N·m), ML classifiers cannot detect bolt loosening; therefore, a 6 N·m torque load decrement was considered the beginning of the early stages of bolt loosening in this investigation.

The motor was fastened by four bolts with 25 N·m torque values that were applied with a torque wrench (Manufacturer: Tohnichi, Model: 450QL3 accuracy: ±3%, Figure 2c), and the motor was operated at a speed of 800 rpm using a motor driver. For generating vibration in the structure, an unbalanced mass (0.25 kg) was fixed to the rotor of the motor. Following this, two accelerometer sensors (Manufacturer: Brüel & Kjær, DK-2830 Virum, Denmark. Type: 4507B004) acquired the vibration signal of the structure for around 30 s and saved the signal on a laptop using an NI cDAQ-9174. The sampling rate of the acquisition system was fixed at 10,000 per second in the experiment, and LabVIEW software was used for recording the data.

Two one-axis accelerometers were used to determine the difference in prediction and detection of the early stages of bolt loosening of the multi-bolt connection system. For the first accelerometer, the axes of the sensor were mounted parallel to the axes of the rotor on the top of the motor, while the second sensor was attached to the back side of the motor, and the axes of the sensor were perpendicular to the axes of the rotor, as shown in Figure 2b. According to supervised learning, the various cases listed in Table 2 were established, and the experiment was performed for all cases, in order to collect enough vibration signals for early stage bolt-loosening detection. In addition, to improve the reliability of the proposed method, vibration signals were also acquired for motor speeds of 1000 rpm and 1200 rpm.

## 4. Results and Discussion

**Figure 3:**
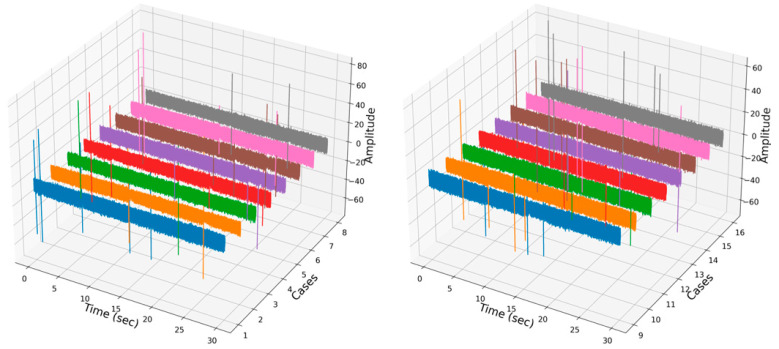
Rows of vibration signals of the dataset corresponding to all cases.

**Figure 4:**
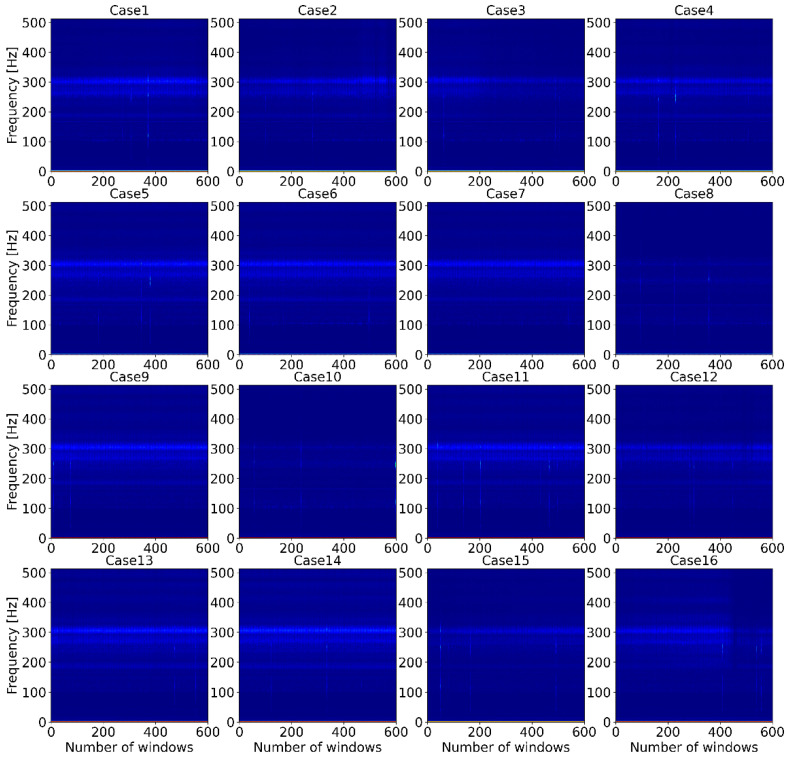
The spectrograms of short-time Fourier transform coefficients for all cases.

As mentioned above, the main goal of this study was to detect the early stages of bolt loosening of the motor and to find the best ML classification algorithm for future work on online monitoring of the motor. Therefore, the various classification algorithms listed in Table 3 were tested in the Python programming language with use of the sklearn package. Figure 3 shows the similarity of the row vibration signal between all cases. The figure shows a row of vibration signals measured by acceleration sensor 2 when the motor operating condition was 800 rpm. All signals were plotted during the timeframe of 30 s. From the figure, it is too complicated to identify or classify which bolt is loosened or not for a human or even an experienced worker. In order to identify key features of the vibration data, STFT was performed, and spectrogram of STFT results are shown in Figure 4. The *Y*-axis of the spectrogram describes 513 FFT coefficients (features), which came from function (3), and the number of segments (windows) lays on the *X*-axis. However, classification of the data, such as finding which spectrogram belongs to which case is still complex, even for experts. However, ML classification algorithms can help to overcome this issue.

**Figure 5:**
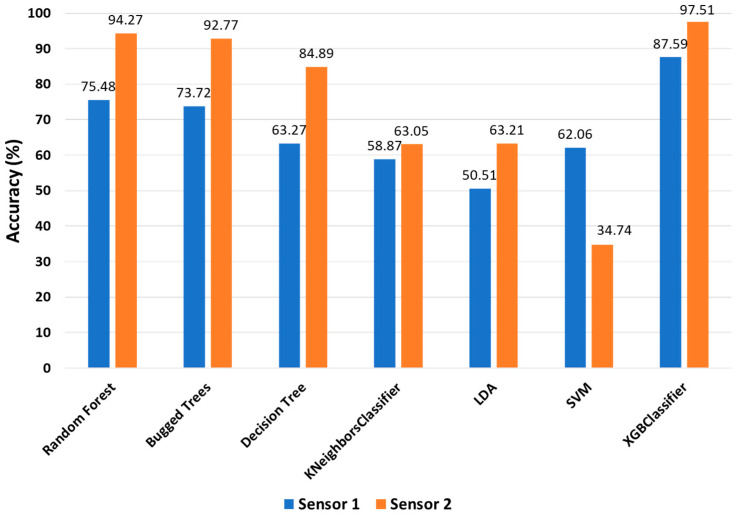
Accuracy of classifiers for both sensors with 513 features.

As already introduced, in order to verify the effect of the accelerometer sensors’ position on the accuracy of the ML classifiers for identification and classification of the early stage of bolt loosening in a multi-bolt connection structure, two sensors were used. Figure 5 shows the accuracy of the ML classifiers, which were used for all features (513 features) for both sensors. All classifiers had a greater accuracy for sensor 2 than sensor 1, except for the SVM classifier. According to the graph, the position of the sensor significantly affected to the accuracy of the classifiers, and the position of sensor 2 was considered the better place for identification and classification of loosening of the multi-bolt connected structure. Therefore, the rest of the results that will be discussed below are for the acceleration signal acquired by sensor 2. It should be noted here that the random forest (RF), bugged trees (BT), and XGBoost classifiers showed an accuracy greater than 90 percent, while the rest of the classifiers performed with lower accuracy.

**Figure 6:**
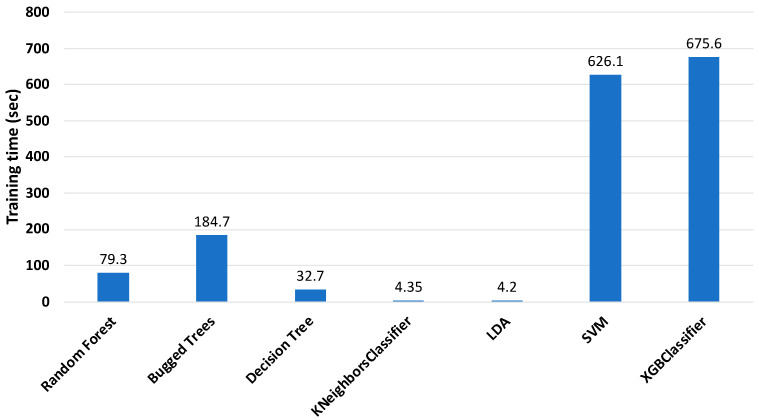
Training time of classifiers with 513 features (sensor 2).

The training times of the classifiers are shown in Figure 6. According to the figure, KNeighbors (KNN) and linear discriminant analysis (LDA) classifiers required the least time for training, which is suitable for online monitoring conditions, but their accuracy was not satisfactory. The RF, BT, and XGBoost classifiers spent too much time for training, despite having the best accuracy. Hence, it was necessary to reduce the training time of the classifiers, while maintaining the accuracy.

**Figure 7:**
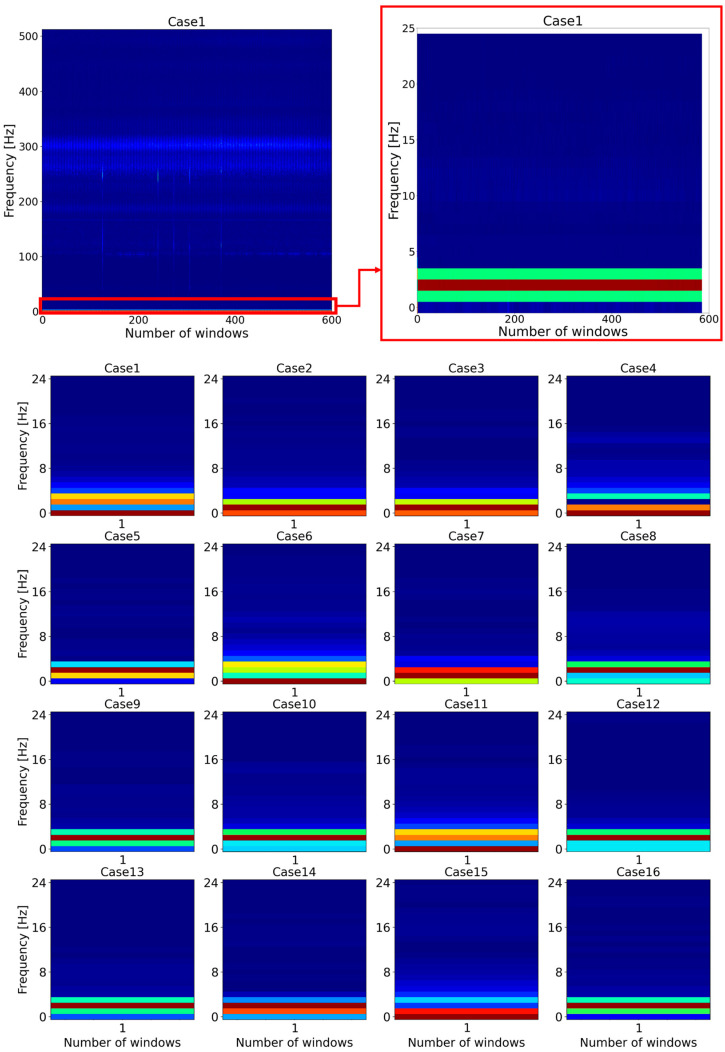
Representation of the feature reduction method for all cases.

To tackle this issue, feature reduction for the dataset was conducted. If the initial spectrogram of STFT results of the vibration signal was zoomed out, the key features of all the data were located between 0 and 5 Hz frequency, as shown in Figure 7. Therefore, the frequencies between 0 and 5 Hz were selected as features. Following that, the classifiers were implemented, but the accuracy of the classifiers decreased significantly. Therefore, the frequency was increased by 5 Hz, and the classifiers were tested. This condition was repeated until satisfactory results were obtained. Consequently, when the frequency was between 0 and 25 Hz, the classifiers had the best accuracy. Following that, in order to represent the differences between all cases more clearly, the spectrograms were plotted for the very beginning window (segment) of all cases.

**Figure 8:**
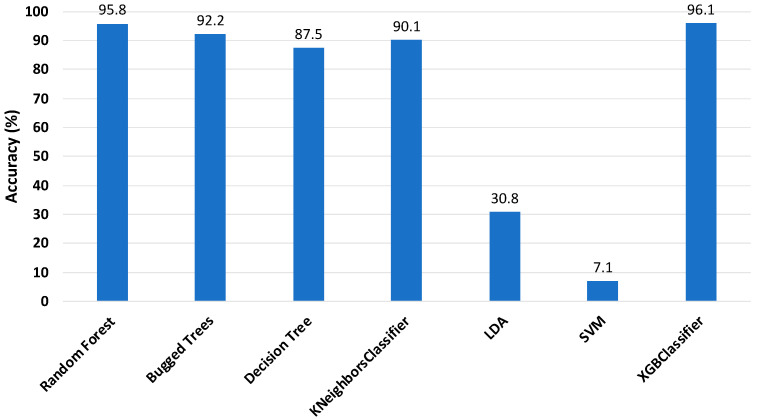
Accuracy of classifiers with 25 features (sensor 2).

Once the feature extraction was performed, all ML classifiers were tested, and the accuracy of the classifiers with 25 features is highlighted in Figure 8. It should be mentioned here that there was a slight increase of around 1.5% in the accuracy of the RF classifier (95.8%), while a slight decrease of approximately 1.5% was observed in the XGBoost classifier accuracy (96.1%). Significant growth was observed in the accuracy of DT and KNN, while there was a dramatic downward trend for LDA and SVM. However, after feature extraction, the BT classifier maintained a stable accuracy (about 92.2%).

**Figure 9:**
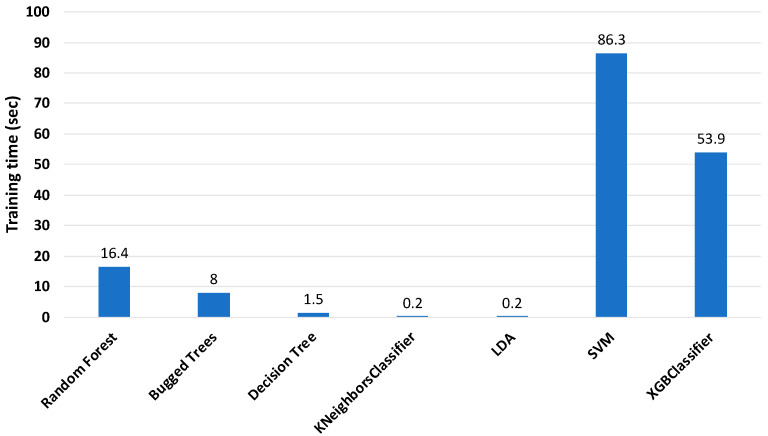
Training time of classifiers with 25 features (sensor 2).

Figure 9 shows the training time of the ML classifiers when the features of the data were reduced from 513 features to 25 features. From the graph, it is clear that there was a large reduction in the training times of the classifiers after feature reduction. As mentioned before, RF and XGboost classifiers had the highest accuracy, of approximately 96%, and their training times were around 16 and 54 s, respectively. According to the results, the RF classifier was selected, with an accuracy around 95.8% and training time of about 16 s, for a future study on the online monitoring of the early stage of loosening of a multi-bolt structure.

The accuracy of a classification is a synthetic variable that can only be used to assess the algorithm’s overall performance, without highlighting any serious concerns about data classification. A confusion matrix can be used to analyze these concerns. A confusion matrix is also known as an error matrix, and it is a representation of statistical classification accuracy. True labels are presented in each row of the confusion matrix, while predicted labels are presented in each column [35].

**Figure 10:**
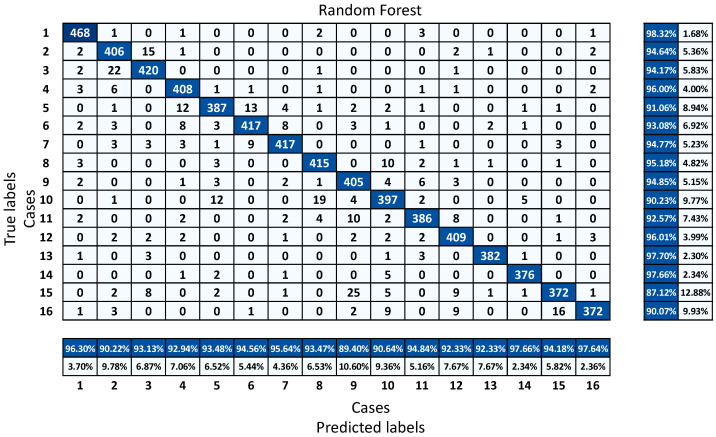
Confusion matrix of RF classifier.

Figure 10 shows the confusion matrix of the RF classifier that was selected as the classifier for a future study. The graph shows detailed information about which labels were predicted correctly and which labels failed in the prediction. In general, case 15 was the hardest label to detect, with 87.1% success, while the simplest case to classify was case 1, with about 98.3% success. Case 5, case 10, and case 16 were also relatively hard to classify for the classifier, with success just above 90%, compared to the rest of the cases.

## 5. Conclusions

In this paper, early stage bolt-loosening detection using ML classifiers for a multi-bolt structure was proposed using a vibration-based method. Then, with the help of STFT, feature extraction was performed on a row of a vibration dataset. Following this, several ML classifiers were trained and evaluated with use of the extracted dataset, to identify the best classifiers for bolt loosening detection on multi-bolt structures.

To reduce computation costs for online monitoring conditions, feature reduction was carried out. The frequencies between 0 and 25 Hz were selected as a feature to obtain a satisfactory accuracy for the ML classifiers. The experimental results showed that the sensor position can affect the accuracy of classifiers for early stage bolt-loosening detection of a multi-bolt structure. From the test results, the RF classifier was selected, with an accuracy around 95.8% and training time of about 16 s, for a future study of online monitoring of loosening of a multi-bolt structure. In addition, the results of the experiments showed that it is not only possible to detect bolt loosening, but also to identify which bolt or bolts start to lose their preload.

This investigation will be broadened by conducting optimization of the sensor position to find the best place for early stage bolt loosening detection and to further develop a RF model to increase the accuracy. The investigation will be applied to online monitoring of a motor in a real condition.

## Figures and Tables

**Table 1:** The specification of AC Servo Motor.

Iteam	Specifications
Name	HIGEN AC Servo Motor
Type	FMATN20-AB00
Capacity	1.8 kW
Torque	11.5 N·m
Max. Speed	1500 rpm
Serial No.	02003001 (ID:74)

**Table 2:** Established necessary cases for bolt-loosening detection and identification.

Case No.	Bolt 1	Bolt 2	Bolt 3	Bolt 4
#1	●	●	●	●
#2	○	●	●	●
#3	●	○	●	●
#4	●	●	○	○
#5	●	●	●	○
#6	○	○	●	●
#7	●	○	○	●
#8	●	●	○	○
#9	○	●	○	●
#10	○	●	●	○
#11	●	○	●	○
#12	○	○	○	○
#13	●	○	○	○
#14	○	●	○	○
#15	○	○	●	○
#16	○	○	○	●

● is tightened and ○ is loosened.

**Table 3:** List of classifiers for bolt-loosening detection.

Number	Classifiers
1	Random Forest
2	Bagged Trees
3	Decision Tree
4	KNeighbor
5	Linear Discriminant Analysis
6	Support Vector Machine
7	XGBoost

## Data Availability

Not applicable.
